# Effectiveness over time of a multimodal intervention to improve compliance with standard hygiene precautions in an intensive care unit of a large teaching hospital

**DOI:** 10.1186/s13756-019-0544-0

**Published:** 2019-05-31

**Authors:** Valentina Baccolini, Valeria D’Egidio, Pasquale de Soccio, Giuseppe Migliara, Azzurra Massimi, Francesco Alessandri, Guglielmo Tellan, Carolina Marzuillo, Corrado De Vito, Marco Vito Ranieri, Paolo Villari

**Affiliations:** 1 Department of Public Health and Infectious Diseases, Sapienza University of Rome, Piazzale Aldo Moro 5, 00185 Rome, Italy; 2 Department of Anesthesiology and Critical Care, Policlinico Umberto I, Sapienza University of Rome, Rome, Italy; 3 Anesthesia and Intensive Care Medicine, Policlinico di Sant’Orsola, Alma Mater Studiorum University of Bologna, Bologna, Italy

**Keywords:** Compliance, Standard hygiene precautions, WHO multimodal strategy, Infection control

## Abstract

**Background:**

Standard hygiene precautions are an effective way of controlling healthcare-associated infections. Nevertheless, compliance with hand hygiene (HH) guidelines among healthcare workers (HCWs) is often poor, and evidence regarding appropriate use of gloves and gowns is limited and not encouraging. In this study, we evaluated the ability over time of a multimodal intervention to improve HCWs compliance with standard hygiene precautions.

**Methods:**

Trend analysis of direct observations of compliance with HH guidelines and proper glove or gown use was conducted in the medical/surgical intensive care unit (ICU) of Umberto I Teaching Hospital of Sapienza University of Rome. The study consisted of two phases: a six-month baseline phase and a 12-month post-intervention phase. The multimodal intervention was based on the World Health Organization strategy and included education and training of HCWs, together with performance feedback.

**Results:**

A total of 12,853 observations were collected from November 2016 to April 2018. Overall compliance significantly improved from 41.9% at baseline to 62.1% (*p* < 0.001) after the intervention and this improvement was sustained over the following trimesters. Despite variability across job categories and over the study period, a similar trend was observed for most investigations. The main determinants of compliance were job category (with nurses having the highest compliance rates), being a member of ICU staff and whether delivering routine, as opposed to emergency, care. HH compliance was modified by glove use; unnecessary gloving negatively affected HH behaviour while appropriate gloving positively influenced it.

**Conclusions:**

The multimodal intervention resulted in a significant improvement in compliance with standard hygiene precautions. However, regular educational reinforcement and feedback is essential to maintain a high and uniform level of compliance.

**Electronic supplementary material:**

The online version of this article (10.1186/s13756-019-0544-0) contains supplementary material, which is available to authorized users.

## Introduction

Adherence to standard hygiene precautions leads to a reduction in infection rates healthcare-associated infections (HAIs) [1, 2], representing the most effective way of preventing cross-transmission of microorganisms [3, 4]. Several economic evaluations show that the promotion of hand hygiene (HH) is a cost-effective intervention, particularly in intensive care unit (ICU) settings [5–7].

However, healthcare worker (HCW) compliance with standard hygiene precautions remains a longstanding challenge. In fact, several studies have highlighted the fact that relatively few HCWs follow correct HH procedures [3, 8], while data on the appropriate use of gloves are more limited but not encouraging [9]. The World Health Organization (WHO) has develop an evidence-based guideline; key for systematic adherence to standard hygiene precautions is education and training of all HCWs, coupled with staff evaluation and performance feedback [4, 10–12]. Moreover, direct observation is recommended as the gold standard for monitoring HCW compliance [13]. Although successful, such WHO strategy has proved that adherence to good practice varies according to the country, local setting, habit, culture and availability of resources [14]. In the Italian context, only a few studies have investigated compliance with HH guidelines [15–17], while a similar number have analyzed changes after educational interventions [14, 18, 19]. Moreover, data on adherence to standard hygiene precautions, which relate to both HH and glove or gown use, are scarce [15]. Furthermore, a detailed long-term assessment of improvements in practice after education is lacking.

The purpose of this study is to evaluate the impact of a multimodal intervention aimed at improving HCW adherence to standard hygiene precautions with an assessment of its effectiveness over time. We tested the hypothesis that focusing on the essential features of HAIs, discussing local evidence of microbial cross-contamination and providing HCWs with education and training on correct procedures of hand hygiene and proper glove or gown use could lead to a substantial behavioural improvement.

## Methods

### Setting

The study was conducted in the medical/surgical ICU of the Umberto I hospital, Sapienza University of Rome, a 1200-bed public hospital. The ICU is divided into five rooms of two beds each, one large seven-bed room and one room for isolation. The ward staff consists of twenty-eight physicians, forty nurses and four healthcare assistants.

### Observation strategy

There are several advantages and disadvantages in determining who will conduct the observations [20]. On the one hand, using infection preventionists require minimum training on standard hygiene precautions guidelines, but it is difficult for them to observe the HCWs without being noticed, resulting in a marked Hawthorne effect. On the other hand, enrolling staff ward promotes widespread acceptance and participation in the activities to improve compliance, even though they might be not completely reliable in rating their colleagues. Therefore, to minimize the Hawthorne effect and to increase staff engagement, we selected as observers the two physicians and three nurses of the ICU that take part in the active surveillance of HAIs that has been carried out in the ICU in collaboration with the Hospital Hygiene Unit of the Umberto I Teaching Hospital since May 2016. In October 2016, at the beginning of the study, they were trained to perform covert observations of compliance with HH guidelines and proper glove or gown use. The training consisted of a two-hour session that included a lecture and an open discussion of the contents of the WHO Hand Hygiene Technical Reference Manual (21) and it was conducted by the resident physicians of the Department of Public Health and Infectious Diseases of Sapienza University of Rome.

For the following two weeks, between 17th and 30th October 2016, the observers were asked to test the usability of an observation form specifically developed to collect data on compliance with standard hygiene precautions and based on the “My Five Moments for Hand Hygiene” approach [21]. Finally, they were invited to discuss together with the trainers the registered observations on 31st October 2016 to compare their data and make as uniform as possible their observation strategy.

From 1st November 2016 to 30th April 2018, the five observers officially monitored their colleagues during daily care activities and collected data using the aforementioned anonymous observation form. The check-sheet focused on four possible types of interaction between HCWs and patients: ‘touching a patient’, ‘device manipulation’, ‘touching patient surroundings’ and ‘invasive procedure or body fluid exposure’. For each type of interaction, the WHO guidelines specifically recommend HH practice both before and afterwards, except for ‘before touching patient surroundings’ that, although it is not strictly mentioned by the WHO, it was included as a relevant opportunity for HH. Additionally, the observers were asked to record glove use during each interaction. For ‘invasive procedure or body fluid exposure’, they also monitored disposable gown wearing. As a result, a total of thirteen different recommendations for standard hygiene precautions were investigated; eight related to HH, four to proper glove use and one to gown use. Both the use of alcohol hand rub and handwashing were part of the HH protocol.

The anonymous form also required the following information: date, day of the week, work shift, observed HCW job category, observed HCW gender, context of delivered care and type of ICU staff. The HCW job categories included physician, nurse, healthcare assistant and other HCW categories (i.e. medical student, technician, therapist).

Throughout the whole study period, the HCWs were aware that they were being observed for compliance with standard hygiene precautions, but they were not told who the observers were or when the observations took place.

The rate of compliance with HH guidelines was measured as the number of HH actions appropriately performed against the total number of opportunities to do so. Since WHO recommends not to use personal protective equipment (such as gloves and gowns) in absence of potential exposure to blood or body fluids [3], glove nonuse was deemed appropriate during ‘touching a patient’ and ‘touching patient surroundings’, in contrast to ‘device manipulation’ and ‘invasive procedure or body fluid exposure’ where glove use was considered appropriate. Similarly, disposable gown wearing ‘during invasive procedure or body fluid exposure’ was considered correct.

The study protocol was approved by the Ethics Committee of the Umberto I Teaching Hospital (reference number: 4707/2017).

### Study design and intervention

The study was made up of two distinct phases; a six-month baseline phase and a 12-month post-intervention phase. From 1st May to 15th May 2017, five identical educational interventions were conducted with the ICU staff to allow all HCWs to take part. During these two-hour sessions, education and training consisted of a lecture on the definition, impact and burden of HAIs, with the first part focusing on major patterns of pathogen transmission and on the critical role of good HH practice and proper glove and gown use in reducing infection rates. The second part of each session presented the results of an active surveillance of HAIs performed during the previous year, giving some evidence of clonal transmission and environmental isolation of some microorganisms (*Acinetobacter baumannii*, *Klebsiella pneumoniae*). In the final part of each session, a targeted feedback on the results of the first six months of this survey was provided to the healthcare personnel to reinforce good practice and specifically address the most critical noncompliance rates. Lastly, since the WHO multimodal strategy outlines the importance of actively engaging HCWs in HH campaigns [4], we encouraged the ICU staff to positively provide peer feedback to their colleagues and motivate them during care activities in order to facilitate awareness-raising about patient safety issues and promote a long-lasting behavioural change.

### Statistical analysis

Observations of compliance with HH guidelines and proper glove or gown use were grouped into six trimesters (two at baseline, four in the post-intervention phase). Descriptive statistics for all variables were calculated. The χ^2^ test was used to compare the average compliance rate in each trimester with respect to the first trimester. A join-point regression was performed to identify time periods with statistically distinct trends (monthly percent change, MPC) in the overall compliance rate over the study period using the Join-point Regression Program, Version 4.6.0.0, National Cancer Institute. The χ^2^ test was also used to compare ‘before’ and ‘after’ indications for HH and to compare HH compliance before and after gloving. Finally, in the univariate analysis, the χ^2^ test was used to assess possible associations between variables and the overall compliance, compliance with HH guidelines and compliance with proper glove or gown use.

Multiple logistic regression models were built to identify factors independently associated with the overall compliance (Model 1), HH compliance (Model 2) and compliance with proper glove or gown use (Model 3). Variables were included in the models when the *p*-value of the univariate analysis was lower than 0.25 or when they were considered relevant to the outcome. As a result, the following variables were used to build the three models: trimester; day of the week; work shift; observed HCW job category; observed HCW gender; type of ICU staff; context of delivered care. In Model 2, the variable indication type (before/after patient contact) was also included. Interaction terms were tested using a significance level cut-off of 0.15. Adjusted OR and 95% confidence intervals (CIs) were calculated.

All statistical analyses were performed with STATA 15 (StataCorp LLC, 4905 Lakeway Drive, College Station, Texas, USA). A *p*-value less than 0.05 was considered statistically significant.

## Results

### Characteristics of recorded observations

Over the 18-month study period, a total of 12,853 observations were collected with a mean of 2142 observations per trimester; of these, 3854 were recorded during the baseline phase and 8999 during the post-intervention phase [see Additional file 1: Table S1].

Observations of compliance with HH procedures accounted for 61.5% of the total with 7908 registered observations. The four types of interaction were similarly represented, with ‘touching a patient’ the most frequently observed (16.5%, 2115 opportunities) and ‘device manipulation’ the least frequently observed (14.1%, 1810 opportunities). ‘Touching patient surroundings’ and ‘invasive procedure or body fluid exposure’ accounted for 15.6 and 15.4%, respectively. By contrast, observations of compliance with proper glove use accounted for 30.8% of the total observations, whereas only 7.7% were of gown use [Additional file 1: Table S1].

The observed staff were largely nursing personnel with 7984 registered observations, accounting for 62.1% of the total; physicians were observed in 4469 cases (34.7%), while only a small number of observations concerned other categories of HCW (2.8%). The observed HCWs were mainly female (63.2%) and members of the ICU staff (88.7%). Most observations were recorded during morning shifts (41.3%), followed by afternoon and night shifts (32.8 and 25.6%, respectively). Almost three-quarters of the observations were performed during week days (73.9%) and the vast majority of observations were of routine care (88.1%) [Additional file 1: Table S1].

### Overall compliance with HH guidelines and proper glove or gown use

**Fig. 1:**
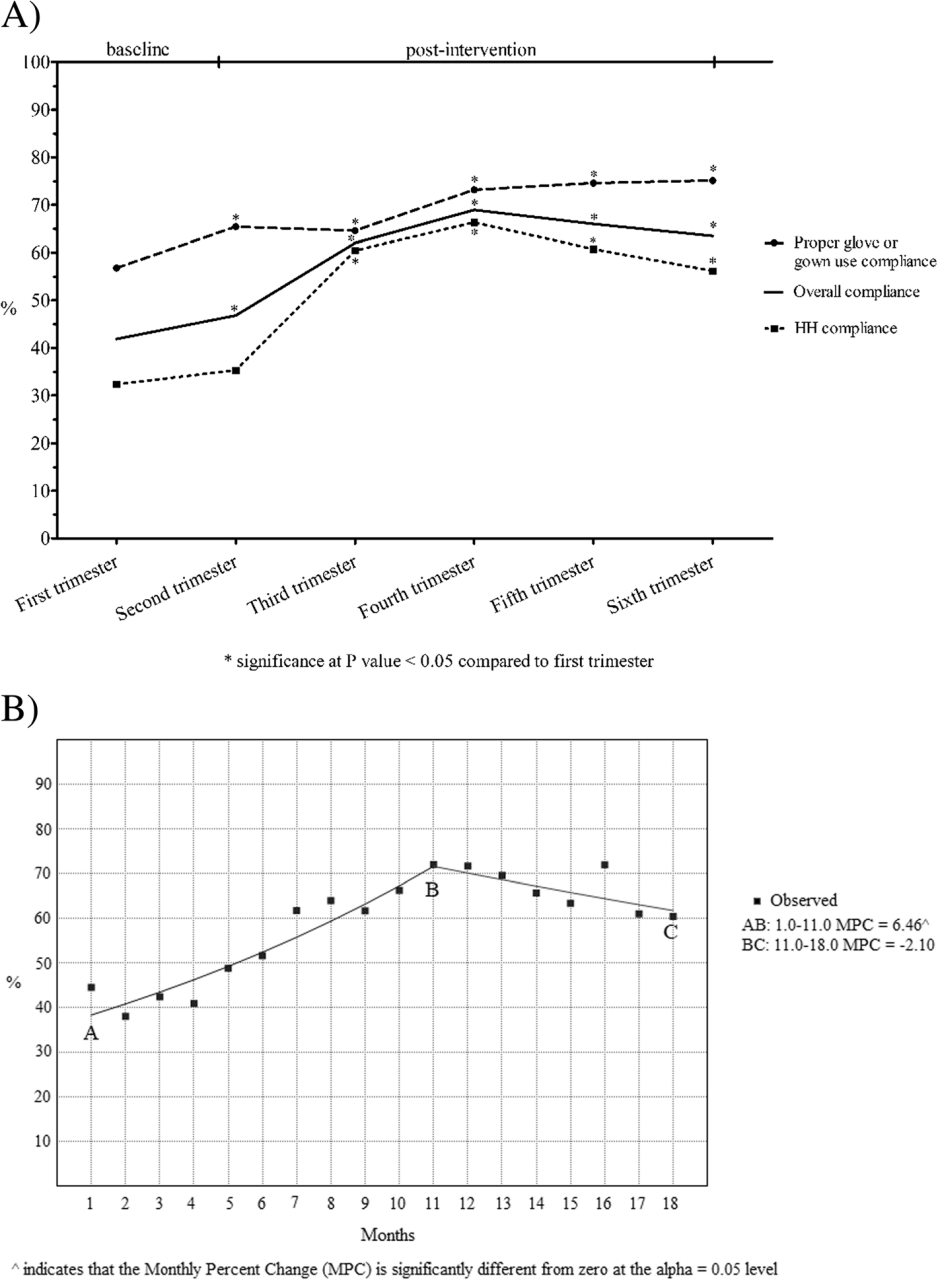
Compliance with standard hygiene precautions over the study period in the intensive care unit of Umberto I Teaching Hospital of Sapienza University of Rome. Results are shown in terms of overall compliance, compliance with hand hygiene (HH) guidelines and compliance with proper glove or gown use over six trimesters (**a**) and in terms of overall compliance in the joinpoint regression (**b**)

After the intervention, the overall compliance rate significantly improved from 41.9% at baseline (first trimester) to 62.1% in the third trimester (*p* < 0.001). This result was maintained during the following three trimesters with an overall compliance rate of 69.0, 66.0 and 63.5% (all comparisons with the first trimester *p* < 0.05). Comparing the first two trimesters at baseline, the overall compliance rate significantly increased from 41.9 to 46.8% (*p* = 0.004), mostly due to the statistically significant increase in proper glove or gown use (56.8% versus 65.5%, *p* = 0.001). Over the 18 months, proper glove- or gown-use compliance was always higher than HH compliance, both at baseline and during the post-intervention phase, with the smallest differences being in the third and fourth trimester [Fig. 1 A].

In the join-point regression analysis, a significant trend variation (MPC: 6.46, *p* < 0.001) in the overall compliance rate was apparent over the first 11 months, while the last 7-month period showed a non-significant variation (MPC: − 2.10, *p* = 0.1) [Fig. 1 B].

### Compliance with HH guidelines

**Fig. 2:**
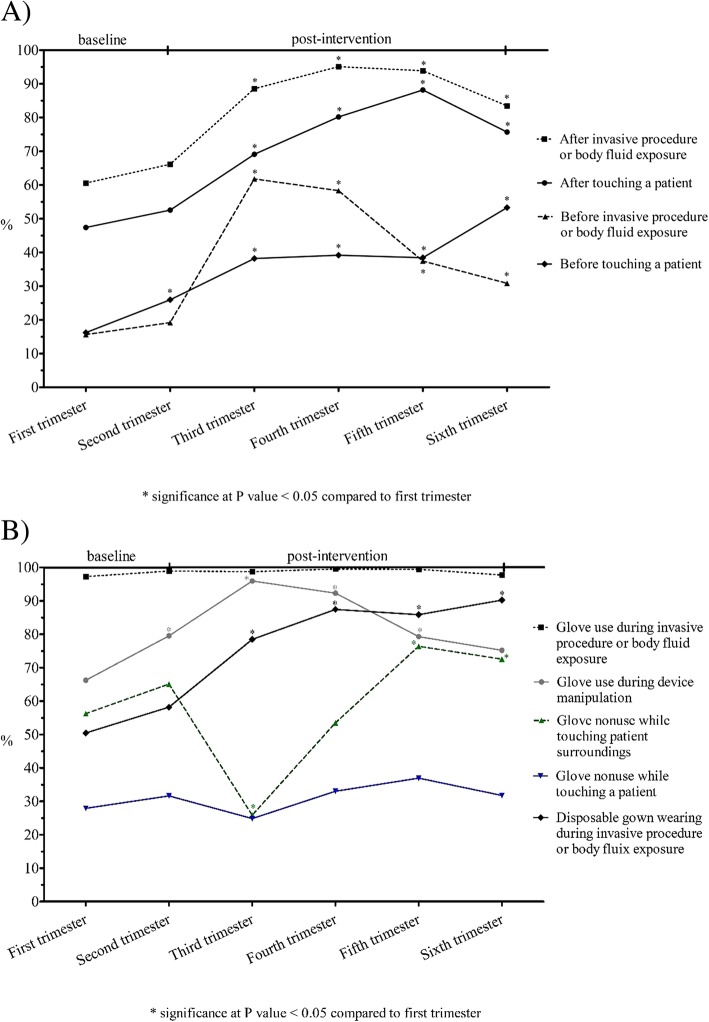
Compliance with standard hygiene precautions over the study period in the intensive care unit of Umberto I Teaching Hospital of Sapienza University of Rome. Results are shown in terms of compliance with hand hygiene guidelines over six trimesters by interaction type (**a**) and in terms of compliance with proper glove or gown use over six trimesters by category (**b**)

Observations of HH practice were analyzed according to the four types of interaction between HCWs and patients. Only those instances concerning ‘touching a patient’ and ‘invasive procedure or body fluid exposure’ are displayed in Fig. 2 A, showing that: i) each compliance rate significantly improved from baseline to post-intervention phase and this result was maintained in the following trimesters (all comparisons with the first trimester: *p* < 0.05); ii) the HH indications before approaching patients (i.e. before ‘touching a patient’ and before ‘invasive procedure or body fluid exposure’) registered lower compliance rates both at baseline and during the post-intervention phase compared to the HH indications after approaching patients (all comparisons: *p* < 0.05). Similar results were obtained for the interaction categories ‘touching patient surroundings’ and ‘device manipulation’ (data not shown).

### Compliance with proper glove or gown use

Compliance with proper glove or gown use was analyzed by category [Fig. 2 B]. Proper glove use ‘during invasive procedure or body fluid exposure’ remained steadily high over time. Proper use of gloves ‘during device manipulation’ significantly improved until a decrease started in the fourth trimester. By contrast, proper nonuse of gloves while ‘touching a patient’ registered low compliance rates throughout the whole study period without showing a significant improvement. Proper glove nonuse while ‘touching patient surroundings’ recorded a significant decrease after the intervention and gradually increased subsequently, with the proper nonuse rates of the last two trimesters significantly higher than baseline*.* Lastly, proper wearing of gowns ‘during invasive procedure or body fluid exposure’ significantly increased after the intervention and this improvement was maintained over time.

### Determinants of compliance

Univariate comparisons revealed no statistically significant differences in the overall compliance rate among shifts (morning: 59.7%, afternoon: 59.9%, night: 58.6%, *p* = 0.47), between weekdays and weekend days (59.4% versus 58.1%, *p* = 0.25) and between male and female HCWs (59.1% versus 59.7%, *p* = 0.48). A statistically significant higher overall compliance rate was found when delivering routine care rather than emergency care (60.2% versus 52.8%, *p* < 0.001) and for internal ICU staff rather than staff external to the unit (60.6% versus 48.0%, *p* < 0.001). With regard to HCW job category, the overall compliance rate for physicians was always lower than the rate for nursing staff, both in the first trimester (40.2% versus 46.0%, *p* = 0.042) and during the first two post-intervention trimesters (54.1% versus 67.1%; 64.9% versus 73.3%, both comparisons *p* < 0.05), but in the last two trimesters the difference was no longer significant (66.2% versus 66.8%, *p* = 0.76; 62.9% versus 64.1%, *p* = 0.64). Similar results were obtained when compliance with HH and proper glove- or gown-use were considered separately; in both univariate analyses, statistically significant higher compliance rates were found for nurses rather than physicians, being internal staff rather than external and delivering routine care rather than emergency care, while the other comparisons did not reveal significant differences (data not shown).

Three multiple logistic regression models were built in order to better investigate the determinants of the overall compliance (Model 1), compliance with HH guidelines (Model 2) and compliance with proper glove or gown use (Model 3) [Table 1].

**Table 1:** Logistic regression models regarding overall compliance (Model 1), compliance with hand hygiene (HH) procedures (Model 2) and compliance with proper glove or gown use (Model 3) over the study period in the Intensive Care Unit (ICU) of the Umberto I Teaching Hospital of Sapienza University of Rome

	Model 1 Overall compliance OR (95%CI)	*P* value	Model 2 HH compliance OR (95%CI)	P value	Model 3 Proper glove or gown use compliance OR (95%CI)	P value
Trimester						
First trimester	Ref.		Ref.		Ref.	
Second trimester	1.17 (1.02–1.35)	0.029	1.12 (0.91–1.37)	0.282	1.34 (1.07–1.69)	0.012
Third trimester	2.29 (1.94–2.70)	< 0.001	4.03 (3.18–5.10)	< 0.001	1.41 (1.08–1.84)	0.013
Fourth trimester	2.92 (2.52–3.39)	< 0.001	4.38 (3.55–5.41)	< 0.001	2.39 (1.87–3.05)	< 0.001
Fifth trimester	2.32 (2.00–2.68)	< 0.001	3.21 (2.62–3.94)	< 0.001	1.99 (1.57–2.54)	< 0.001
Sixth trimester	2.08 (1.78–2.43)	< 0.001	2.53 (2.03–3.15)	< 0.001	2.13 (1.63–2.76)	< 0.001
Day						
Week day	Ref.		Ref.		Ref.	
Weekend day	1.10 (0.99–1.22)	0.064	1.13 (0.98–1.30)	0.106	1.10 (0.92–1.32)	0.282
Work shift						
Morning	Ref.		Ref.		Ref.	
Afternoon	1.07 (0.97–1.17)	0.184	0.95 (0.83–1.08)	0.400	1.32 (1.12–1.55)	0.001
Night	0.93 (0.84–1.03)	0.144	0.82 (0.72–0.95)	0.006	1.08 (0.91–1.29)	0.354
Observed healthcare worker (HCW) job category						
Physician	Ref.		Ref.		Ref.	
Nurse	1.23 (1.12–1.34)	< 0.001	1.18 (1.04–1.33)	0.008	1.38 (1.19–1.60)	< 0.001
Healthcare assistant	0.18 (0.12–0.26)	< 0.001	0.09 (0.05–0.15)	< 0.001	0.26 (0.16–0.45)	< 0.001
Other	0.36 (0.24–0.52)	< 0.001	0.19 (0.11–0.35)	< 0.001	0.57 (0.31–1.06)	0.076
Observed HCW gender						
Female	Ref.		Ref.		Ref.	
Male	0.99 (0.92–1.08)	0.944	0.96 (0.86–1.07)	0.496	1.05 (0.91–1.21)	0.521
Observed ICU staff						
External	Ref.		Ref.		Ref.	
Internal	1.61 (1.39–1.87)	< 0.001	2.55 (2.05–3.18)	< 0.001	1.14 (0.89–1.45)	0.289
Observed care context						
Emergency care	Ref.		Ref.		Ref.	
Routine care	1.64 (1.42–1.88)	< 0.001	2.17 (1.77–2.65)	< 0.001	1.38 (1.10–1.74)	0.006
Indication type						
Before patient contact	–		Ref.		–	
After patient contact	–		5.43 (4.86–6.08)	< 0.001	–	

In the first model, the overall compliance significantly increased after the first trimester, with the highest OR in the fourth (*p* < 0.001). Overall compliance was also positively associated with being a nurse rather than a physician (*p* < 0.001), being an internal staff member rather than external (*p* < 0.001) and when delivering routine care rather than emergency care (*p* < 0.001). By contrast, being a healthcare assistant or another HCW job category was negatively associated with the outcome (both *p* < 0.001). Finally, day of the week, work shift and observed HCW gender did not show a significant association with the overall compliance. Similar associations were found in the second and third model. In Model 2, multivariate analysis confirmed the results of Model 1 with the exception of work shift, where working at night negatively affected compliance with HH guidelines (*p* = 0.006). Additionally, HH indications after patient contact were found to be the strongest determinants of the outcome (OR: 5.43, 95%CI: 4.86–6.08). In Model 3, the results were comparable with the first model with a few exceptions: being an internal staff member or another HCW job category did not correlate with proper glove or gown use, while working during morning shifts was negatively associated with compliance [Table 1].

HH compliance was modified by glove use. In particular, unnecessary gloving negatively affected HH behaviour while appropriate gloving positively influenced it. Indeed, both before and after approaching patients, HH compliance rates were significantly lower when HCWs wore gloves incorrectly for ‘touching a patient’ and ‘touching patient surroundings’ (all comparisons: *p* < 0.05). By contrast, HH compliance rates before and after necessary gloving were significantly higher both during ‘device manipulation’ and ‘invasive procedure or body fluid exposure’ if HCWs wore gloves (all comparisons: *p* < 0.05) [see Additional file 1: Table S2].

## Discussion

This study consisted of two phases, a baseline phase and a post-intervention phase, during which direct observations of compliance with standard hygiene precautions were recorded. The implementation of a multimodal intervention led to a significant compliance improvement across all types of HH indications and most glove- or gown-use observations, with a mean compliance increase comparable with other post-intervention studies [22]. In line with other findings [23, 24], compliance with most recommendations reached a peak after which performance began to decline. This highlights the difficulty in maintaining a high rate of adherence to recommended practice over time and the importance of providing educational reinforcement and performance feedback to HCWs so that improvements can be sustained [25]. In most cases, decreases in compliance began between the fourth and sixth trimester, suggesting that repeating the intervention within twelve months of the first implementation could maximize its effectiveness over time, as proposed by van de Mortel et al. [26]. Interestingly, we also observed a statistically significant compliance increment in the second trimester of the baseline phase, which is probably due to the observer effect*,* while the improvement in the second trimester of the post-intervention phase might be due to a combination of peer monitoring and peer feedback. As shown in other studies [27, 28], such increase may confirm that approaches creating an environment that promote and motivate HCWs to change can contribute to a more effective and lasting improvement in compliance.

Compliance varied across professional categories and in relation to several factors. Nurses started with and achieved the highest level of compliance, both in general and with HH recommendations, as reported by the vast majority of other studies [8, 25, 29]. Physician compliance was always lower than that of nursing staff but registered a greater increase after the intervention. Conversely, healthcare assistants and other HCW categories were associated with lower compliance rates.

Other factors appeared to negatively affect compliance, including being external to the ICU and delivering care during emergency situations, the latter of which is probably due to the fact that HH in these situations is perceived as a waste of time [30]. Factors such as day of the week and gender of the observed HCW did not influence the compliance rates. Notably, the negative impact of working at night on HH compliance and the adverse association between working during morning shifts and compliance with proper glove or gown use may be related to either the observer effect or peer monitoring and feedback. Particularly, they may have been less emphasized during night shifts, resulting in a significant decrease in HH compliance, and overrepresented during daily activities, when they led to an inappropriate overuse of personal equipment. For these reasons, other training sessions will be conducted with the HCWs to further address the correct indications for compliance with both HH and proper glove or gown use.

For each HH indication, compliance significantly increased after the intervention, but compliance remained highest for interactions that took place after approaching patients rather than before, in line with other studies [8, 25, 31]. Interestingly, while for most investigations HH adherence gradually peaked after the intervention, compliance with HH practices ‘before invasive procedure or body fluid exposure’ decreased immediately after the third trimester; as shown in other studies [9, 32], this drop may be due to the hard to change wrong perception that HH is not necessary prior to wearing gloves.

With regard to gloves or gown recommendations, we observed that compliance with their proper use was greater than compliance with HH indications, as reported by Pan et al. [15]. Furthermore, in the investigations where gloving or gowning was required, the intervention was effective in significantly improving such compliance rates, except for ‘glove use during invasive procedure or body fluid exposure’ where HCWs were already compliant at baseline. By contrast, for those interactions where gloving was unnecessary, the intervention did not bring about a significant steady increase in appropriate use. Actually, we registered an inappropriate glove overuse both for ‘while touching patient surroundings’ and ‘while touching a patient’ immediately after the intervention, which probably means that the educational sessions were not effective in addressing the correct indications for glove use and HCWs have preferred gloving, even if unnecessary.

Notably, the impact of wearing gloves on adherence to HH guidelines was twofold. On the one hand, although glove use is not a substitute for HH [3], inappropriate glove use adversely affected HH compliance. This may be due to the erroneous belief that glove use alone is sufficient to limit the spread of microorganisms and therefore it obviated the need for good HH practice. On the other hand, proper glove use was positively associated with hand disinfection as shown in other studies [9, 15]. Therefore, since inappropriate glove overuse might contribute to poor HH compliance, further HCW education of proper glove use is required. Particularly, customized training courses focusing on the consequences of unnecessary use of gloves will be scheduled with the HCWs in order to promote their correct use and investigate the behavioural determinants of inappropriate overuse.

Despite the significant improvements reported, HH compliance rates did not reach a uniform and optimal level of adherence. As indicated by previous studies [33–35], to interrupt cross-transmission of microorganisms in settings at high risk of infection, good HH practice needs to be performed in at least 60–80% of the situations where it is required. The heterogeneity we observed in compliance rates (either over time, or between staff) might be responsible for the lack of significant reduction in HAIs after the intervention, as registered by the ICU surveillance system. In fact, incidence rates of device-related infections remained higher than the 90th percentile (as defined in the National Healthcare Safety Network report [36]) throughout the study period, in line with other Italian incidence rates [37], without a clearly decreasing trend and with some evidence of clonal transmission of microorganisms (data not shown). Therefore, since no other change or intervention was registered in the ICU over the 18 months, we believe that the mean HH compliance rate of 61.6% that we found suggests the need to further implement effective measures in order to achieve higher compliance rates and obtain clinical benefits such as a reduction in HAIs.

This study has some strengths and limitations. The main strength is the comprehensive evaluation of adherence to standard hygiene precautions and the changes in compliance rates over the ensuing trimesters. We also distinguished HH indications according to the type of interaction between HCWs and patients. Additionally, we were able to correlate compliance rates with incidence of HAIs in the same ICU over time. The limitations of our research are mostly due to the use of direct observation to monitor HCW behaviour. Even though HCWs did not know who the observers were and which practices were recorded, compliance data may be influenced by the observer effect. Moreover, enrolling HCWs from the ICU to collect data and perform the observations might have made them inclined to rate their coworkers differently than outside observers would. Differences among observers might also have affected accuracy. However, the impact of these biases was probably limited by the large number of observations over time and the random selection of practices, as recommended by the WHO [3]. Lastly, since we did not assign unique identifiers to each HCW, we could not compare compliance before and after the intervention at an individual level. Such a comparison could be an interesting area for future investigations to further study individual predictors and factors that contribute to compliance.

## Conclusions

Despite variability across HCW job categories and types of recommendation, the multimodal intervention was effective in improving compliance with standard hygiene precautions over time. However, since in the vast majority of the investigations the compliance started to decline between six and twelve months after the educational intervention, providing a tailored reinforcement of indications and procedures for good HH practice within one year after its first implementation is advisable to achieve and maintain a uniform and high level of adherence. Additionally, given that glove use seemed to significantly influence compliance with HH practices, promoting strategies to reduce misuse and overuse of gloves are needed.

## Additional file

Additional file 1: **Table S1.** Characteristics of recorded observations over the study period concerning compliance with hand hygiene (HH) guidelines and proper glove or gown use in the Intensive Care Unit (ICU) of the Umberto I Teaching Hospital of Sapienza University of Rome. **Table S2.** Compliance with hand hygiene (HH) procedures by indication in relation to glove use over the study period in the intensive care unit of the Umberto I Teaching Hospital of Sapienza University of Rome. (DOCX 25 kb)

## Data Availability

The datasets used and/or analysed during the current study are available from the corresponding author on reasonable request.

## Abbreviations

HAI: Healthcare associated infection
HCW: Healthcare worker
HH: Hand hygiene
ICU: Intensive care unit
WHO: World Health Organization
