# Supplementary material for: Effectiveness over time of a multimodal intervention to improve compliance with standard hygiene precautions in an intensive care unit of a large teaching hospital
Source: Antimicrob Resist Infect Control. 2019 May 31;8:92. doi: 10.1186/s13756-019-0544-0 (PMC6544958; doi:10.1186/s13756-019-0544-0)
Supplement: Supplementary file 1 — Table S1. Characteristics of recorded observations over the study period concerning compliance with hand hygiene (HH) guidelines and proper glove or gown use in the Intensive Care Unit (ICU) of the Umberto I Teaching Hospital of Sapienza University of Rome. Table S2. Compliance with hand hygiene (HH) procedures by indication in relation to glove use over the study period in the intensive care unit of the Umberto I Teaching Hospital of Sapienza University of Rome. (DOCX 25 kb) [file 13756_2019_544_MOESM1_ESM.docx]

**Additional file 1**

**Table S1**

|  | | | Total  N (%) |  | Baseline phase | |  | Post-intervention phase | | | |  |
| --- | --- | --- | --- | --- | --- | --- | --- | --- | --- | --- | --- | --- |
|  |  |  |  |  | First trimester  N (%) | Second trimester  N (%) |  | Third  trimester  N (%) | Fourth trimester  N (%) | Fifth  trimester  N (%) | Sixth  trimester  N (%) |  |
|  | | |  |  |  |  |  |  |  |  |  |  |
| Observation category | | | 12853 (100) |  | 1282 (100) | 2572 (100) |  | 2013 (100) | 2842 (100) | 2481 (100) | 1663 (100) |  |
|  | Hand hygiene observations | | 7908 (61.5) |  | 782 (61.0) | 1588 (61.7) |  | 1236 (61.4) | 1746 (61.4) | 1536 (61.9) | 1020 (61.3) |  |
|  |  | Before or after touching a patient | 2115 (16.4) |  | 308 (24.0) | 433 (16.8) |  | 330 (16.4) | 424 (14.9) | 406 (16.4) | 214 (12.9) |  |
|  |  | Before or after device manipulation | 1810 (14.1) |  | 160 (12.5) | 354 (13.8) |  | 298 (14.8) | 416 (14.6) | 348 (14.0) | 234 (14.0) |  |
|  |  | Before or after touching patient surroundings | 2004 (15.6) |  | 96 (7.5) | 424 (16.5) |  | 294 (14.6) | 460 (16.2) | 424 (17.1) | 306 (18.4) |  |
|  |  | Before or after invasive procedure or body fluid exposure | 1979 (15.4) |  | 218 (17.0) | 377 (14.6) |  | 314 (15.6) | 446 (15.7) | 358 (14.4) | 266 (16.0) |  |
|  | Glove and gown use observations | |  |  |  |  |  |  |  |  |  |  |
|  |  | Glove use | 3956 (30.8) |  | 391 (30.5) | 795 (30.9) |  | 619 (30.8) | 873 (30.7) | 768 (31.0) | 510 (30.7) |  |
|  |  | Disposable gown wearing | 989 (7.7) |  | 109 (8.5) | 189 (7.4) |  | 158 (7.9) | 223 (7.9) | 177 (7.1) | 133 (8.0) |  |
| Observed healthcare worker (HCW) job category | | | 12853 (100) |  | 1282 (100) | 2572 (100) |  | 2013 (100) | 2842 (100) | 2481 (100) | 1663 (100) |  |
|  | Nurse | | 7984 (62.1) |  | 622 (48.5) | 1679 (65.3) |  | 1205 (59.9) | 1774 (62.4) | 1649 (66.5) | 1055 (63.4) |  |
|  | Physician | | 4469 (34.7) |  | 604 (47.1) | 813 (31.6) |  | 730 (36.3) | 948 (33.4) | 778 (31.4) | 596 (35.8) |  |
|  | Healthcare assistant | | 227 (1.8) |  | 43 (3.4) | 52 (2.0) |  | 46 (2.3) | 68 (2.4) | 15 (0.6) | 3 (0.2) |  |
|  | Other | | 125 (1.0) |  | 9 (0.7) | 19 (0.7) |  | 6 (0.3) | 52 (1.8) | 33 (1.3) | 6 (0.4) |  |
|  | Missing | | 48 (0.4) |  | 4 (0.3) | 9 (0.3) |  | 26 (1.3) | 0 (0.0) | 6 (0.2) | 3 (0.2) |  |
| Observed HCW gender | | | 12853 (100) |  | 1282 (100) | 2572 (100) |  | 2013 (100) | 2842 (100) | 2481 (100) | 1663 (100) |  |
|  | Female | | 8127 (63.2) |  | 859 (67.0) | 1782 (69.3) |  | 1179 (58.6) | 1695 (59.6) | 1463 (59.9) | 1149 (69.1) |  |
|  | Male | | 4570 (35.6) |  | 423 (33.0) | 784 (30.5) |  | 753 (37.4) | 1110 (39.1) | 997 (40.2) | 503 (30.2) |  |
|  | Missing | | 156 (1.2) |  | 0 (0.0) | 6 (0.2) |  | 81 (4.0) | 37 (1.3) | 21 (0.9) | 11 (0.7) |  |
| Observed ICU staff | | | 12853 (100) |  | 1282 (100) | 2572 (100) |  | 2013 (100) | 2842 (100) | 2481 (100) | 1663 (100) |  |
|  | Internal | | 11396 (88.7) |  | 1068 (83.3) | 2296 (89.3) |  | 1594 (79.2) | 2511 (88.4) | 2361 (95.2) | 1566 (94.2) |  |
|  | External | | 1122 (8.7) |  | 204 (15.9) | 183 (7.1) |  | 317 (15.7) | 245 (8.6) | 98 (4.0) | 75 (4.5) |  |
|  | Missing | | 335 (2.6) |  | 10 (0.8) | 93 (3.6) |  | 102 (5.1) | 86 (3.0) | 22 (0.9) | 22 (1.3) |  |
| Work shift | | | 12853 (100) |  | 1282 (100) | 2572 (100) |  | 2013 (100) | 2842 (100) | 2481 (100) | 1663 (100) |  |
|  | Morning | | 5307 (41.3) |  | 467 (36.4) | 1103 (42.9) |  | 701 (34.8) | 1299 (45.7) | 927 (37.4) | 810 (48.7) |  |
|  | Afternoon | | 4219 (32.8) |  | 356 (27.8) | 797 (31.0) |  | 927 (46.1) | 870 (30.6) | 805 (32.4) | 464 (27.9) |  |
|  | Night | | 3295 (25.6) |  | 459 (35.8) | 653 (25.4) |  | 376 (18.7) | 673 (23.7) | 749 (30.2) | 385 (23.2) |  |
|  | Missing | | 32 (0.3) |  | 0 (0.0) | 19 (0.7) |  | 9 (0.4) | 0 (0.0) | 0 (0.0) | 4 (0.2) |  |
| Day | | | 12853 (100) |  | 1282 (100) | 2572 (100) |  | 2013 (100) | 2842 (100) | 2481 (100) | 1663 (100) |  |
|  | Week day | | 9496 (73.9) |  | 879 (68.6) | 1875 (72.9) |  | 1314 (65.3) | 1965 (69.1) | 2039 (82.2) | 1424 (85.6) |  |
|  | Weekend day | | 2044 (15.9) |  | 403 (31.4) | 541 (21.0) |  | 114 (5.7) | 305 (10.7) | 442 (17.8) | 239 (14.4) |  |
|  | Missing | | 1313 (10.2) |  | 0 (0.0) | 156 (6.1) |  | 585 (29.1) | 572 (20.1) | 0 (0.0) | 0 (0.0) |  |
| Observed care context | | | 12853 (100) |  | 1282 (100) | 2572 (100) |  | 2013 (100) | 2842 (100) | 2481 (100) | 1663 (100) |  |
|  | Routine care | | 11318 (88.1) |  | 1132 (88.3) | 2191 (85.2) |  | 1530 (76.0) | 2522 (88.7) | 2359 (95.1) | 1584 (95.2) |  |
|  | Emergency care | | 1427 (11.1) |  | 147 (11.5) | 360 (14.0) |  | 470 (23.3) | 308 (10.8) | 79 (3.2) | 63 (3.8) |  |
|  | Missing | | 108 (0.8) |  | 3 (0.2) | 21 (0.8) |  | 13 (0.6) | 12 (0.4) | 43 (1.7) | 16 (1.0) |  |

**Table S1**. Characteristics of recorded observations over the study period concerning compliance with hand hygiene (HH) guidelines and proper glove or gown use in the Intensive Care Unit (ICU) of the Umberto I Teaching Hospital of Sapienza University of Rome.

**Table S2**

|  | | HH not performed  N (%) | HH performed  N (%) |  | HH not performed  N (%) | HH performed  N (%) |  | HH not performed  N (%) | HH performed  N (%) |  | HH not performed  N (%) | HH performed  N (%) |  |
| --- | --- | --- | --- | --- | --- | --- | --- | --- | --- | --- | --- | --- | --- |
|  | | Before touching a patient ** | |  | Before touching patient surroundings * | |  | Before device manipulation ** | |  | Before invasive procedure or body fluid exposure *** | |  |
| Gloving | |  |  |  |  |  |  |  |  |  |  |  |  |
|  | Not performed | 456/725 (62.9) | 269/725 (37.1) |  | 221/403 (54.8) | 182/403 (45.2) |  | 113/150 (75.3) | 37/150 (24.7) |  | 11/12 (91.7) | 1/12 (8.3) |  |
|  | Performed | 239/332 (72.0) | 93/332 (28.0) |  | 435/599 (72.6) | 164/599 (27.4) |  | 478/754 (63.4) | 276/754 (36.6) |  | 590/977 (60.4) | 387/977 (39.6) |  |
|  | |  | |  |  | |  |  | |  |  | |  |
|  | | After touching a patient * | |  | After touching patient surroundings * | |  | After device manipulation * | |  | After invasive procedure or body fluid exposure ** | |  |
| Gloving | |  |  |  |  |  |  |  |  |  |  |  |  |
|  | Not performed | 188/726 (25.9) | 538/726 (74.1) |  | 105/403 (26.0) | 298/403 (74.0) |  | 82/150 (54.7) | 68/150 (45.3) |  | 6/12 (50.0) | 6/12 (50.0) |  |
|  | Performed | 139/332 (41.9) | 193/332 (58.1) |  | 306/599 (51.1) | 293/599 (48.9) |  | 150/754 (19.9) | 604/754 (80.1) |  | 163/978 (16.7) | 815/978 (83.3) |  |
|  | * Significance at P value < 0.001 | | |  |  |  |  |  |  |  |  |  |  |
|  | ** Significance at P value < 0.01 | | |  |  |  |  |  |  |  |  |  |  |
|  | *** Significance at P value < 0.05 | | |  |  |  |  |  |  |  |  |  |  |

**Table S2.** Compliance with hand hygiene (HH) procedures by indication in relation to glove use over the study period in the intensive care unit of the Umberto I Teaching Hospital of Sapienza University of Rome.
